# Adverse childhood experiences and disengagement from HIV care: a case-cohort study in Tanzania

**DOI:** 10.1186/s12981-025-00760-6

**Published:** 2025-06-27

**Authors:** Sydney Tucker, Solis Winters, Emmanuel Katabaro, Matilda Mlowe, Patrick Bradshaw, Jennifer Ahern, John Colford, Laura Packel, Susan Hillis, Amon Sabasaba, Prosper Njau, Sandra I. McCoy

**Affiliations:** 1 University of California, Berkeley, United States; 2 University of Oxford, Oxford, United Kingdom; 3 Health for a Prosperous Nation, Dar es Salaam, United Republic of Tanzania; 4 Imperial College London, London, United Kingdom; 5 Ministry of Health, Dodoma, United Republic of Tanzania

**Keywords:** Adverse childhood experiences, HIV, Disengagement from care, Antiretroviral therapy, Tanzania

## Abstract

Adverse childhood experiences (ACEs) can have lasting, detrimental effects throughout the lifespan and may influence engagement in health care. We conducted a case-cohort study in Tanzania to estimate the association between ACEs and disengagement from HIV care 12 months after initiating antiretroviral therapy (ART) among 217 adults (26 cases and 191 sub-cohort participants). Experiencing one, two, three, and four additional ACEs was significantly associated with 28% (RR_a_= 1.24; 95% CI: 1.05, 1.44; *p*-value < 0.01), 64% (RR_a_=1.64; 95% CI: 1.22, 2.20), 110% (RR_a_=2.10; 95% CI: 1.35, 3.26), and 168% (RR_a_=2.68; 95% CI: 1.49, 4.38) increases in the risk of disengagement from HIV care, respectively. These findings call for integrated trauma-informed mental health services within HIV care to end HIV/AIDS as a public health threat.

## Background

There are over 1.7 million people living with HIV (PLHIV) in Tanzania [1], more than any other country in Eastern Africa, which makes the country a global priority for preventing and treating HIV. Antiretroviral therapy (ART) can interrupt transmission and reduce both morbidity and mortality; however, its success hinges on high levels of adherence to achieve viral suppression. The 2023 UNAIDS report announced that Tanzania achieved target goals of > = 95% of PLHIV knowing their status, >=95% of people who know their status on ART, and > = 95% of people on treatment achieving viral suppression [1]. While achieving the ’95-’95-’95 targets is a major public health milestone, sustaining these gains remains a critical challenge. Active planning to sustain ‘95-95-95’ achievements and protect these decades of progress is essential, as evidence shows that sustaining long-term adherence to treatment is a pivotal hurdle requiring targeted, strategic support [2, 3]. Importantly, ART adherence underpins all three ’95-’95-’95 targets, making it fundamental to sustaining epidemic control [4]: treatment adherence yields viral suppression, which simultaneously prevents onward transmission. Therefore, increased attention on supporting PLHIV who have the greatest barriers to treatment adherence is highly strategic. Now is the time to identify and mitigate key, measurable, and actionable barriers threatening long-term retention in HIV care to sustain ’95-’95-’95 targets and reach UNAIDS goals of eliminating HIV as a public health threat by 2030 [2]. 

Adverse Childhood Experiences (ACEs), defined as exposures to abuse, violence, and household and community challenges experienced prior to age 18, may influence suboptimal engagement in HIV care and poor adherence to ART. ACEs are both highly prevalent among PLHIV [5] and have been linked to > 40 negative health outcomes [6], many of which are associated with ART treatment interruptions, including increases in depression, anxiety, psychological distress, alcohol and drug use, unintended pregnancy, future violence victimization, and increased risk-taking behavior [7, 8]. It is therefore plausible to hypothesize that adverse childhood experiences may measurably influence disengagement in HIV care, a pathway potentially mediated through interim outcomes like mental health, substance use, gender-based violence in adulthood, and increased engaged in risky behaviour [5, 9]. 

Although there is a growing body of literature on the high prevalence of ACEs among PLHIV and the potential for trauma to influence HIV care adherence [10], there is a lack of evidence on the direct, quantitative association between ACEs and disengagement from HIV care, ART adherence, and/or viral suppression, especially in low and middle income countries (LMICs). To date, the majority of research on ACEs and adherence to HIV care have occurred in high-income countries [11], and studies in LMICs have often focused on only one individual ACE, such as child sexual abuse [12], or on traumatic experiences more broadly, such as gender-based violence occurring anytime across the lifespan [13]. Despite strong associations between ACEs and negative health outcomes that are linked to sub-optimal adherence to HIV care [7, 8], evidence examining the direct association between ACEs and adherence to HIV care in LMICs remains sparse. We therefore conducted a case-cohort study nested within a randomized controlled trial [14] in Lake Zone, Tanzania to investigate the association between ACEs and disengagement from HIV care– in a country with a critically high prevalence of both HIV and ACEs [1, 15]. 

The aims of this case-cohort study are: (1) estimate the prevalence of ACEs among adult PLHIV in Lake Zone, Tanzania; (2) assess whether there is an association between cumulative number of ACEs and disengagement from HIV care; (3) explore if present mental health challenges may be a mediator between ACEs and disengagement from HIV care. We hypothesized that an increase in ACE score would be associated with an increased risk of disengagement from HIV care among PLHIV in Tanzania.

## Methods

### Parent study

To assess our hypothesis, we conducted a case-cohort study nested within a cluster randomized controlled trial (“parent study”) evaluating the impact of short-term economic support on viral suppression across four regions (Mwanza, Shinyanga, Geita and Kagera) in Lake Zone, Tanzania (clinicaltrials.gov identifier: NCT04201353) [14]. In the parent study, participants were recruited at 32 HIV clinics (8 clinics per region), among adult PLHIV (aged 18 and older) who initiated ART within the last 30 days. Trial participants (*n* = 1990) were followed for the primary outcome of viral suppression at 12-months (endline). This nested case-cohort study is reported following the Strengthening the Reporting of Observational Studies in Epidemiology (STROBE) guidelines [16]. 

### Rationale for case-cohort design

ACEs are sensitive and distressing topics to discuss– which can result in triggering memories for participants with ACEs. Therefore, collecting ACEs data requires additional training for surveyors to minimize distress, prevent triggers, respond sensitively, respect privacy, and make mental health referrals for participants if needed. To protect participants and reduce the time required from clinic staff conducting the surveys, we aimed to ask ACE-related survey questions to as few participants as possible. Thus, we selected a case-cohort design for the gains in statistical efficiency compared to a traditional cohort design and the benefit that the odds ratio calculated from this design directly estimates the risk ratio, without a rare disease assumption [17]. 

### Study population and eligibility

On September 24, 2022, we identified 317 trial participants who met two inclusion criteria: (1) enrolled in the parent study between August 14, 2021 and October 5, 2021 and would therefore have reached 11–13 months on ART, ensuring that participants were either approaching or had just reached trial endline and 12-months of follow-up; and (2) had yet to complete the parent trial’s 12-month survey, where ACE information would be collected. From the parent trial [14]we had access to outcome data for all trial participants; however, exposure data on ACEs was only measured among participants sampled into the case-cohort study– through adding an ACE module into the parent trial’s 12-month endline survey, conducted in Swahili by HIV clinic staff (in-person or over the phone). Clinic staff were given training on sensitivity and privacy when asking potentially triggering questions, and they offered all participants mental health referrals.

#### Case definition

Cases were individuals who disengaged from HIV care at 12 months after initiating ART, defined as > 90 days elapsing since their last scheduled clinic appointment anytime during the window of 11–13 months after trial enrollment, based on medical record review. Though recent PEPFAR guidelines define interruption in HIV treatment as no clinical contact for 28 days after the last scheduled appointment, 28 days has been widely used as an indicator of “lateness”, while > 90 days has been widely used as a threshold to represent true disengagement [18]. While the threshold of 28 days is crucial for programmatic purposes of increasing tracing practices to help PLHIV return to care promptly to avoid long ART interruptions, we use the definition of > 90 days to be consistent with the widespread threshold representing a longer period of disengagement from HIV care– to identify PLHIV who are disengaged from care and may have higher risk of both accelerated disease progression and onward HIV transmission. Additionally, we did not include in our case definition participants who missed a scheduled clinic appointment by > 90 days but then returned to care prior to 12 months, as the outcome was disengagement from care *without return*: the group perceived to be most at-risk of unsuppressed viral load and transmission to others. Case status was determined through medical record data, including both biometric mHealth system data and manually abstracted medical file data.

#### Sub-cohort sampling

Consistent with a case-cohort design, we randomly selected a sub-cohort of participants from the sampling frame to represent the control group, regardless of their outcome status. In the sampling frame of *n* = 317 there were 83 people who met the case definition; thus, we randomly selected a sub-cohort of *n* = 250 to enable a case: sub-cohort ratio of 1:3. Cases and sub-cohort members were not matched.

#### Case sampling

Following case-cohort study methodology, we included all cases. There were 83 cases: 63 of whom met the case definition in the randomly selected sub-cohort of 250, and the remaining 20 cases were sampled additionally. In total, the study included 270 participants. Researchers were masked to exposure status when identifying case status.

### Recruitment

We contacted all sampled individuals for their endline survey through at least 3 phone calls, 3 text messages, and 3 home visits through home-based care providers, who trace individuals who have disengaged from HIV care according to Ministry of Health guidelines in Tanzania. Participants who were verified as having transferred to another clinic via official clinic records were excluded, because we could not access appointment data at their transfer clinics to verify current care status.

### Exposure measurement

We adapted the Adverse Childhood Experiences International Questionnaire (ACE-IQ) [19] to assess ACE exposure, which is a validated scale created for collecting data on ACEs in LMIC. ACE-IQ has been used extensively globally, especially in Africa and even specifically in Tanzania [20]. Our survey assessed the first 10 categories within the ACE-IQ scale which were relevant to the Tanzanian context: (1) physical abuse; (2) emotional abuse; (3) sexual abuse; (4) emotional neglect; (5) household violence; (6) parental death or divorce; (7) household drug abuse; (8) household member incarceration; (9) household mental illness; (10) community violence. We used the binary ACE-IQ scale, which collects data on whether participants ever experienced ACEs, not frequency of occurrence. For all questions, participants are asked to report on events they experienced *prior* to the age of 18, which would have occurred *before* they initiated ART and enrolled into our parent study, thus ensuring the exposure preceded the outcome. A participant’s ACE score is the summation of ACE categories they experienced (range 0–10) and is treated as a continuous variable in all analyses.

### Statistical analysis

We first described the prevalence of all individual ACEs in our sub-cohort. Next, we described the mean and median number of ACEs experienced among participants in our sample, stratified by cases and sub-cohort members. To assess the relationship between ACEs and disengagement from HIV care, we initially fit an unadjusted logistic regression model to estimate odds ratios; when applying logistic regression to the case cohort design, the estimated odds ratios represent the relative risk of disengagement from HIV care at 12 months associated with a one-unit increase in ACE score [17]. 

For our primary analysis, we adjusted the model for potential confounders of sex, age, and region, as well as arm of the parent trial, to ensure our results are not influenced by the intervention designed to reduce disengagement from ART. Based on our primary model, we also used the beta estimate from the exposure variable (ACE score) and standard error to estimate the adjusted risk of disengagement from HIV care associated with experiencing two-, three-, and four-unit increases in ACE score. We explored using a cubic term, linear spline, quadratic spline, and restricted quadratic spline to model our continuous exposure variable; however, when using Bayesian Information Criterian (BIC) and Akaike Information Criterion (AIC) to compare these models, the more parsimonious model using a simple linear term for ACE score was favored (smallest BIC and AIC). Consistent with a case-cohort approach, [17]all participants sampled in the sub-cohort who developed the outcome were included in our sample twice: once in the sub-cohort and once as a case. Therefore, we use robust standard errors in all models to account for individual clustering.

Next, we applied inverse probability weighting to account for survey non-response [21]. Stabilized weights were estimated from predicted probabilities of missingness, based on logistic regression results regressing survey non-response on variables collected in the parent trial’s baseline survey, including both standard covariates and outcomes, which may be correlated with ACE score such as mental health, stigma, and perceived social status. Weights were then incorporated into the primary model described above. Confidence intervals were estimated through 1,000 bootstrap iterations, where we maintained the original ratio of cases, sub-cohort members without the outcome, and sub-cohort members with the outcome across all resampling iterations– then extracted the 0.975 and 0.025 quantiles. We used R for all data cleaning and data analysis.

Finally, we conducted an additional exploratory analysis controlling for mental health– using Patient Health Questionnaire 2 (PHQ-2) as a measure of depression and Generalized Anxiety Disorder (GAD-2) as a measure of anxiety– to estimate the association between adverse childhood experiences and disengagement from HIV care extending beyond the association between mental health difficulties and disengagement from HIV care. PHQ and GAD are both commonly used to measure mental health and have been specifically used among PLHIV in Tanzania [22, 23]. The purpose of this analysis is: (1) explore whether the pathway between ACEs and disengagement from HIV care is mediated by current mental health challenges; (2) investigate whether screening for ACEs would provide additional benefit in identifying PLHIV at-risk of disengagement from HIV care, beyond solely screening for mental health (as is currently used in some HIV clinics). For this analysis, we first constructed models controlling for mental health reported 12-months after ART initiation, at the timepoint when disengagement from HIV care was measured; then, we constructed separate models to control for mental health reported at ART initiation.

## Results

After tracing 270 eligible participants, we found that 5 participants died, 5 participants transferred to another facility and were excluded, and 60 participants could not be reached despite our robust tracing plan. Thus, we received endline surveys from 191 participants from our randomly sampled sub-cohort (76% response rate) and 26 of 73 cases (36% response rate, after exclusion of 10 participants who died or transferred). Of the 191 participants in the sub-cohort, 17 developed the outcome; consistent with a case-cohort approach, these participants appeared in the analytical dataset twice: once in the sub-cohort and once as a case. The final analytical sample was *n* = 217 (*n* = 191 in the sub-cohort and *n* = 26 cases).

**Fig. 1:**
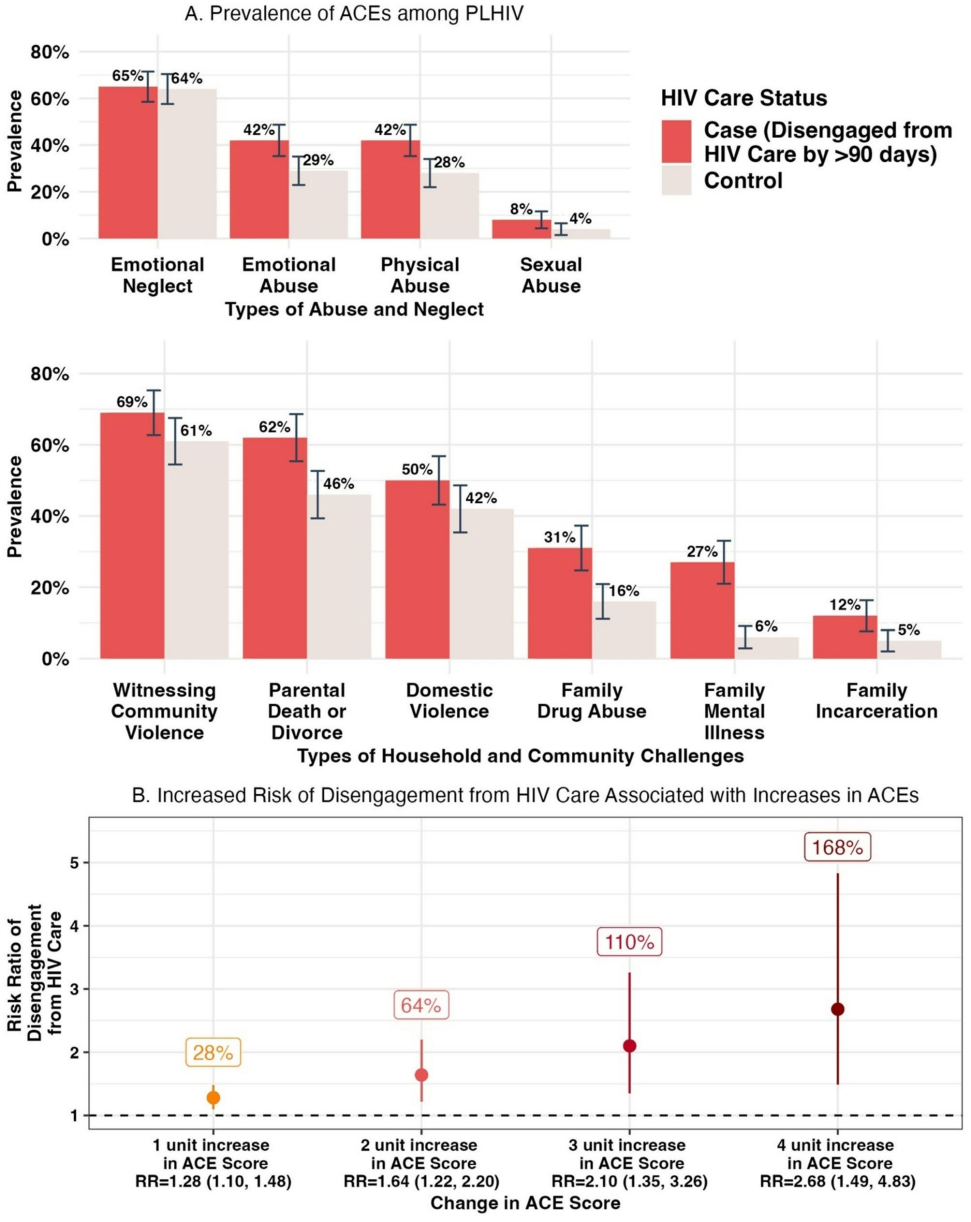
Prevalence of Retrospectively Reported Adverse Childhood Experiences (ACEs) among Adults Living with HIV in Laze Zone, Tanzania by HIV Care Status (**A**), and Change in Risk of Disengagement from HIV Care in Adulthood Associated with Increases in ACEs (**B**) in 2022–2023

Within the sub-cohort of 191 participants (mean age = 37; 62% female), the most prevalent ACEs were emotional neglect (65%), witnessing community violence (61%), parental death or divorce (46%), witnessing family violence (42%), and experiencing emotional abuse (29%) and physical abuse (28%). The vast majority experienced at least one ACE (96%), and 35% experienced four or more ACEs. Comparing the sub-cohort population to the case population, mean ACE scores were 2.88 (standard deviation [SD] = 1.93) and 4.8 (SD = 2.62) among the sub-cohort (*n* = 191) and cases (*n* = 26), respectively. All ACEs were more prevalent among cases, with two times the prevalence of sexual abuse (8% vs. 4%) and a family member who experienced mental illness (27% vs. 6%), drug abuse (31% vs. 16%), or past incarceration(s) (12% vs. 5%) among cases compared to sub-cohort participants, respectively (Fig. 1). There were also marked increases in experiences of physical abuse, emotional abuse, and parental death or divorce, comparing cases to the sub-cohort (Fig. 1). Among participants who experienced 0 ACEs (*n* = 8), 1–3 ACEs (*n* = 131), and 4 + ACEs (*n* = 78), 0%, 12%, and 17% of participants had disengaged from HIV care at 12 months, respectively.

Unadjusted results revealed that a one-unit increase in ACE score is associated with 1.24 (95% CI: 1.05, 1.44; *p*-value < 0.01) times the risk of disengagement from HIV care at 12-months after ART initiation, defined as > 90 days since the last scheduled appointment. Adjustment for age, sex, region, and parent trial arm yielded consistent results (RR_a_=1.28; 95% CI: 1.10, 1.48; *p*-value = 0.02) (Fig. 1). Using this model, two-unit, three-unit, and four-unit increases in ACE score were associated with a significant increase in the risk of disengagement from HIV care at 12 months by 64% (RR_a_=1.64; 95% CI: 1.22, 2.20), 110% (RR_a_=2.10; 95% CI: 1.35, 3.26), and 168% (RR_a_=2.68; 95% CI: 1.49, 4.38), respectively (Fig. 1). Crucially, adjusted results modeling the relative risk of disengagement from HIV care associated with a one-unit change in ACE score remained consistent after incorporation of stabilized weights from inverse probability weighting to account for our survey response rate (RR_w_=1.24; 95% CI: 1.07, 1.42). Next, when controlling for mental health challenges 12-months after ART initiation, results did not change (RR_a_=1.25; 95% CI: 1.06, 1.47; *p*-value = 0.005). Finally, when controlling for mental health challenges at ART initiation, the risk ratio remained stable with only a slight move away from the null (RR_a_=1.30; 95% CI: 1.11, 1.53; *p*-value = 0.001).

## Discussion

Adverse childhood experiences were highly prevalent among people living with HIV in Lake Zone, Tanzania: The average number experienced was almost 3 among PLHIV and almost 5 among PLHIV disengaged from HIV care. All ACEs were experienced more often among participants who had disengaged from HIV care at 12 months after starting ART (cases), compared to the sub-cohort of PLHIV. Higher levels of ACEs were significantly associated with meaningful increases in the risk of disengagement from HIV care; for example, experiencing three- and four-unit higher ACE scores more than doubled the likelihood of disengagement from HIV care. This study provides preliminary evidence that ACEs are highly prevalent and may meaningfully increase the risk of disengagement from lifesaving, transmission-preventing HIV care among PLHIV in Tanzania.

Our findings contribute to the small, growing body of literature examining the prevalence of ACEs among PLHIV and the direct association between ACEs and disengagement from HIV care. The direction of our results, along with the high prevalence of ACEs specifically in Tanzania, is consistent with Whetton et al.’s research in 2013, which is the only known study examining adverse childhood experiences and self-reported disengagement from HIV care in Tanzania [24]. Our results are additionally consistent with qualitative findings from Enane et al. which described potential mechanisms linking trauma and disengagement from HIV care among adolescents in Kenya [22]. Our findings differ from studies that did not find a significant association between ACEs and disengagement from HIV care; for example, in a systematic review of childhood sexual abuse (CSA), other ACEs, and ART adherence among adults, only 4 of 8 studies reported significantly harmful relationships between CSA or other ACEs and ART adherence [6]. However, only 1 of these 8 studies was conducted outside of high-income countries, where ACE prevalence is often lower than in LMICs. Furthermore, while research in high-income countries has yielded inconsistent results on ACEs and suboptimal ART adherence, there remains a growing discussion about the potentially substantial impacts of the syndemics of trauma and HIV [10]. This timely study is among the first to examine the quantitative association between a variety of ACEs and disengagement from HIV care in a LMIC utilizing medical record data to define disengagement from HIV care, rather than relying on self-report, using a rigorous and efficient case-cohort design.

Future researchers should examine this important question in larger samples, with increased focus on the associations between specific ACEs and suboptimal ART adherence, as well as thorough investigation of potential mediating pathways. ACEs related to childhood sexual abuse and family member(s) experiencing mental illness, drug use, and/or incarceration history had the largest differences in prevalence between cases and sub-cohort participants in our sample, which hints that these ACEs may be the strongest drivers of our results. Additionally, thorough exploration of the causal pathways between ACEs and disengagement from HIV care is needed. Based on our analysis, mental health does not appear to be a key mediator; the association between ACEs and disengagement from HIV care 12-months after ART initiation was unchanged after controlling for mental health at the same 12-months timepoint, indicating mental health does not explain the association between ACEs and disengagement from HIV care. Existing evidence of the association between ACEs and increased risk-taking behavior, substance use, and victimization of violence in adulthood– combined with evidence linking these exposures to disengagement from HIV care– suggests that these variables should be explored as potential mediators on the pathway between ACEs and disengagement from HIV care. Additionally, our results were stable after controlling for mental health at ART initiation, which indicates that ACEs may predict disengagement from HIV care beyond the prediction provided via mental health screenings at ART initiation. This data suggests that screening for ACEs among new ART initiates, as has been piloted in the United States [25], could be considered to enable early identification and linkage to supportive resources for clients who may be most at-risk of disengagement from HIV care. Estimating associations between individual ACEs and disengagement from HIV care, along with identifying causal mechanisms, will be especially important to inform targeted screenings and interventions to improve ART adherence.

Our study has important limitations. The survey response rate among cases was suboptimal (36%); this low response rate is related to difficulties locating cases despite repeated attempts, due to their out-of-care status, which may introduce selection bias into our study. However, to address potential selection bias, we incorporated stabilized weights from inverse probability weighting to account for missingness by ‘up-weighting’ participants with similar characteristics to those who are missing, and the results remained consistent; if our weighting variables fully capture the reasons for loss to follow-up, this provides evidence that the association between ACEs and disengagement from ART would remain if we had a higher follow-up rate. In addition, we did not have sufficient statistical power to explore the association between *individual* ACEs and disengagement from HIV care, nor the data to assess differences in results accounting for frequency of exposure to ACEs, which are both especially important for identifying PLHIV who may be most at-risk of disengagement from HIV care and building targeted programming. As with observational studies, we may have uncontrolled or residual confounding; for example, we expect that poverty in early childhood (*prior* to any ACEs) may be an important yet unmeasured confounder. Lastly, ACEs are appropriately based on self-report. Although we expect that recall of these events is very high, as traumatic experiences tend to be remembered throughout one’s lifetime, it is possible that some participants were not comfortable sharing ACEs with clinic staff or research staff who conducted the surveys, and this may lead to an underreporting of ACEs. We have no reason to suspect underreporting would differ by case status; however, if this difference exists, we hypothesize it is more likely that cases underreported ACEs, since they are not engaged in care and may therefore have less of a trusting relationship with clinic staff who conducted the surveys compared to participants who are in care– which would bias the findings towards the null.

## Conclusion and implications

These findings add to existing calls to galvanize utilization of funding and resources to integrate trauma-informed mental health initiatives within HIV care to eliminate HIV/AIDS as a public health threat by 2030. Screening for adverse childhood experiences may be a tool to improve early identification and linkage to supportive resources for clients who may be most at-risk of disengagement from HIV care; especially in Tanzania, where intimate partner violence screenings and resource referral systems are already utilized in many clinics, questions related to ACEs could be easily integrated. Lastly, crucially, while mental health care programming has increased within certain HIV settings in recent years, the majority of these programs do not incorporate elements dedicated to supporting PLHIV to cope with previous traumatic exposures experienced in childhood and learn skills to build resilience. Our results suggest the value in further exploration of whether expanding existing mental health programming to specifically facilitate recovery from childhood adversity may strengthen the effectiveness of these programs on improvements in ART adherence, to eliminate HIV/AIDS as a public health threat by 2030.

## Data Availability

Individual de-identified participant data that underlie these results can be made available upon request. Proposals should be directed to the senior author; to gain access, data requestors will need to sign a data access agreement and show evidence that the proposed use of the data has been approved by an independent ethical review committee identified for this purpose.
